# “It’s still a great adventure” – exploring offshore employees’ working conditions in a qualitative study

**DOI:** 10.1186/s12995-017-0179-0

**Published:** 2017-12-08

**Authors:** Janika Mette, Marcial Velasco Garrido, Volker Harth, Alexandra M. Preisser, Stefanie Mache

**Affiliations:** Institute for Occupational and Maritime Medicine, University Medical Centre Hamburg-Eppendorf, Hamburg, Seewartenstr. 10, 20459 Hamburg, Germany

**Keywords:** Offshore wind industry, Working conditions, Job demands, Job resources, Qualitative analysis

## Abstract

**Background:**

Despite the particular demands inherent to offshore work, little is known about the working conditions of employees in the German offshore wind industry. To date, neither offshore employees’ job demands and resources, nor their needs for improving the working conditions have been explored. Therefore, the aim of this study was to conduct a qualitative analysis to gain further insight into these topics.

**Methods:**

Forty-two semi-structured telephone interviews with German offshore employees (*n* = 21) and offshore experts (*n* = 21) were conducted. Employees and experts were interviewed with regard to their perceptions of their working conditions offshore. In addition, employees were asked to identify areas with potential need for improvement. The interviews were analysed in a deductive-inductive process according to Mayring’s qualitative content analysis.

**Results:**

Employees and experts reported various demands of offshore work, including challenging physical labour, long shifts, inactive waiting times, and recurrent absences from home. In contrast, the high personal meaning of the work, regular work schedule (14 days offshore, 14 days onshore), and strong comradeship were highlighted as job resources. Interviewees’ working conditions varied considerably, e.g. regarding their work tasks and accommodations. Most of the job demands were perceived in terms of the work organization and living conditions offshore. Likewise, employees expressed the majority of needs for improvement in these areas.

**Conclusions:**

Our study offers important insight into the working conditions of employees in the German offshore wind industry. The results can provide a basis for further quantitative research in order to generalize the findings. Moreover, they can be utilized to develop needs-based interventions to improve the working conditions offshore.

**Electronic supplementary material:**

The online version of this article (10.1186/s12995-017-0179-0) contains supplementary material, which is available to authorized users.

## Background

Offshore wind is a relatively new technology that has become competitive with other forms of energy in Europe [1]. Among European countries, Germany is considered one of the pioneers in the offshore wind energy sector. In recent years, the German offshore wind industry has increased rapidly [2], leading to a substantial growth in employment figures. In 2015, approximately 20,500 persons worked in the German offshore wind industry [3]. According to rough estimates, the number of direct and indirect workers in this sector is expected to increase to 33,000 by 2021 [4].

### Working conditions offshore

It has been described that working in the German offshore wind industry places high demands on its employees, in terms of personal, technical, and safety-related qualifications [5, 6]. Offshore employees in Germany perform their work in the hazardous work environment of the high seas. Their work schedule generally consists of 2 weeks offshore work, followed by 2 weeks free time onshore (i.e. 14/14-work schedule). During their offshore assignments, employees typically work for 12 hours a day [7–11]. They must often perform their tasks under time pressure [9] and are exposed to unfavourable weather conditions that might pose risks to themselves and cause work delays or interruptions [7, 8, 10]. Working at heights or on the deck of the platforms often places the workers in physically demanding and potentially dangerous situations [6, 7, 10]. Furthermore, the workers’ transportation from the platforms to the offshore wind farms has been described as challenging [8, 10], and the remote location of the wind parks represents an obstacle in case of emergencies [7, 9, 10]. Depending on the type of offshore environment, employees must work in confined spaces, share double cabins, and may experience limited privacy [7–9, 11]. Moreover, they might be subjected to a restricted social environment and must deal with recurrent periods of absence from home [7, 9, 11].

Despite these considerations for German offshore workers being outlined in single reports and statements, empirical data on the topic is still missing. To our knowledge, no empirical research studies have been carried out that systematically examine the working conditions of employees in the growing German offshore wind branch. However, closing this research gap and gaining a deeper understanding of their perceived work situation is highly relevant, especially since offshore work has been described as particularly challenging and stressful [12, 13].

Research studies from related branches, e.g. the international wind industry, offshore oil and gas industry, and seafaring branch [7, 14], have also revealed high job demands for their workers, including high quantitative demands [15, 16], perceived risks at work [17], harsh environmental conditions [18], and long absences from home [17, 19–22]. Nonetheless, transferring the findings from these industries to the German offshore wind branch is not permissible; despite sharing certain similarities, there remain distinctive differences between the industries, e.g. regarding the workflows, rules and regulations [7]. This makes an in-depth investigation of the working conditions in the German offshore wind branch necessary. Due to the lack of available data, a qualitative research approach was chosen to gain first explorative insights into the topic.

### Theoretical background

When exploring offshore employees’ working conditions, the Job Demands-Resources model [JD-R model] by Demerouti and Bakker [23, 24] provides an adequate theoretical framework. The JD-R model was introduced as a further development to other relevant models, such as the Job Demands-Control model [25] and the Effort-Reward-Imbalance model [26]. It incorporates a broad range of working conditions for which it describes two key categories: job demands (aspects of the job that require physical or mental effort and are associated with certain physiological and psychological costs) and job resources (aspects of the job that may assist in achieving work goals, reduce job demands and the associated costs, and stimulate personal growth) [23, 24].

### Study aims

The aim of our study was to conduct a qualitative analysis to gain further insight into offshore employees’ perceived working conditions, and to identify both hindering and conducive factors of offshore work from their perspective. Furthermore, we intended to compare and contrast the perceptions of offshore employees and experts, and to investigate offshore employees’ needs for improvement regarding their work. We addressed the following research questions:

1. *What are offshore employees’ and experts’ perceptions of demands and resources of offshore work?*
2. *What are offshore employees’ specific needs for improvement regarding their working conditions?*

## Methods

### Participants

Data were gathered through 42 semi-structured telephone interviews with offshore workers (*n* = 21) and offshore experts (*n* = 21) during July and August 2016. The qualitative interview approach was chosen as it seemed most suitable to acquire initial knowledge on the topic. Due to practicability and logistical issues, all interviews were conducted via telephone. To be eligible, both offshore workers and experts had to be over the age of 18 and had to be fluent in German. Furthermore, the offshore employees had to work in a regular 14/14-work schedule. The sample of offshore experts was restricted to persons with professional expertise, work experience, and managerial responsibility (e.g. health and safety specialists, occupational physicians, offshore managers, and managers of offshore service providers). Participation in the study was on a voluntary basis. For announcing the interview study, invitation emails were sent and leaflets were distributed to human resources departments and company physicians of offshore wind energy operators via mail, asking them to promote the interview study and recruit suitable participants. Additionally, we presented the study in health and safety trainings for offshore workers, encouraging the workers to participate in the study and to inform their colleagues and supervisors. Prior to the interviews, a written informed consent was sent to and signed by all participants. The telephone interviews were carried out by the first author who had prior experiences with conducting qualitative interviews. A pre-test interview was performed, and feedback to the interviewer was provided by supervisors and fellow members. Interview length ranged from 24 min (expert) to 1 hour (employee). All interviews were conducted in German and were tape recorded.

### Interview guideline

The interviews were conducted using two slightly varying versions of a semi-structured interview guideline (one for the employees and one for the experts). The guideline was developed in accordance with the research questions and was based on the theoretical framework (JD-R model [23, 24]). The interview topic list is depicted in Table 1 in the order in which the topics are presented in the guideline. Different questions regarding employees’ and experts’ perceptions of their working conditions and employees’ needs for improvement were integrated. The interview guideline was piloted with the help of an offshore worker and minor revisions were made based on the worker’s recommendations.

**Table 1:** Interview topic list

**Introduction**	study information, confidentiality, informed consent
**Socio-demographics**	offshore occupation, offshore experience in years
	*employees:* age, relationship status
**Working conditions**	job demands *(example question: “When you think about your offshore work, what are particular [physical / psychological] demands of your work?”)*
	job resources *(example question: “When you think about your offshore work, what do you particularly like about your work?”)*
	*employees:* needs for improvement *(example question: “In general, what needs for improvement do you see regarding your working conditions offshore?”)*

### Analysis

Audio recordings from the interviews were transcribed and anonymized. The transcripts were analysed by the first author in a deductive-inductive process according to Mayring’s qualitative content analysis, using the software MAXQDA Analytics Pro (version 12, VERBI GmbH, 2016). Different codes, categories, and sub-categories were systematically identified and refined in an iterative process to fit the data. The results were discussed thoroughly in several meetings with all co-authors. Precisely, discrepancies were reviewed and differences were resolved by in-depth discussions until consensus was reached. Reflexivity (e.g. regarding the researchers’ personal values, objectives and preconceptions, and how they might affect the research) was enhanced at every step of the research process. For the purpose of publication, interviewees’ quotes were translated into English with the support of an English native speaker.

## Results

### Participants

Of the 21 offshore employees we interviewed, 19 (90.5%) were male and 2 (9.5%) were female (Table 2). With respect to the offshore experts, 17 (81.0%) were male and 4 (19.0%) were female. Employees’ average experience in the offshore wind industry was 3.4 years (range 7 months – 8 years), whereas experts’ average experience was 5.1 years (range 6 months – 10 years). 10 (47.6%) employees and 17 (81.0%) experts had at least 3 years of offshore experience. Almost one third (28.6%) of the employees were technicians. 11 (52.4%) experts worked as health and safety managers and 4 (19.0%) as occupational physicians. 11 (52.4%) employees were aged between 31 and 40 years. 18 (85.7%) employees were in a relationship.

**Table 2:** Participant characteristics (*n* = 42)

Variable	Employees (*n* = 21)		Experts (*n* = 21)	
	*n*	%	*n*	%
**Gender**				
male	19	90.5	17	81.0
female	2	9.5	4	19.0
**Offshore experience**				
< 1 years	2	9.5	3	14.3
1 − 2 years	9	42.9	1	4.8
3 − 4 years	5	23.8	7	33.3
> 4 years	5	23.8	10	47.6
	Mean: 3.4 years Range: 7 months – 8 years		Mean: 5.1 years Range: 6 months – 10 years	
**Occupation**				
technician	6	28.6	–	–
quality and maintenance	5	23.8	–	–
paramedic	3	14.3	–	–
health and safety	3	14.3	11	52.4
management offshore	4	19.0	3	14.3
occupational physician	–	–	4	19.0
managing director of offshore service providers	–	–	3	14.3
**Age**				
20 − 30 years	5	23.8	n.a.	n.a.
31− 40 years	11	52.4	n.a.	n.a.
41− 50 years	4	19.0	n.a.	n.a.
> 50 years	1	4.8	n.a.	n.a.
**Relationship status**				
relationship	18	85.7	n.a.	n.a.
single	3	14.3	n.a.	n.a.

### Offshore employees’ working conditions

**Fig. 1:**
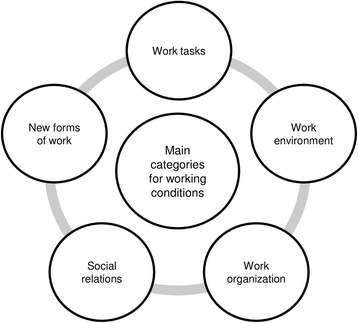
Main categories for offshore employees’ working conditions

The job demands and resources that emerged from the interviews can be summarized into the five main categories depicted in Fig. 1.

In addition to the main categories, we identified various associated sub-categories. All main categories and their related sub-categories are depicted in tables and elaborated below. In particular, central aspects are further expanded upon in the next paragraphs. A table with all main and sub-categories can be found in the Additional file 1.

### Category 1: Work tasks

All job demands and resources named in terms of the category ‘work tasks’ are depicted in Table 3.

**Table 3:** Job demands and resources in terms of the work tasks

Main category: Work tasks	
**Job demands**	
physical work	hard physical work
transfer and access to installations	physically and psychologically challenging
accident risks	e.g. danger of falling or slipping
workloads	intermittent periods of high workloads
demanding work tasks	demanding work tasks that require high concentration
work equipment	heavy work tools and personal protective equipment
**Job resources**	
meaning of work	associations: adventure, freedom, fulfilment, pioneer spirit
perception of safety	high perception of safety, strict safety procedures
motivation and satisfaction	high work motivation and satisfaction
challenging work tasks	positively challenging work tasks
skills and competencies	work requires high skills and competencies
versatility of work	great versatility of the work
scope of action	wide scope of action at work

### Job demands

#### Physical work

The physical work was considered demanding by all workers and experts:

*“Generally, the work is very physically taxing.” [employee, ID #17, age 31–40 years, offshore experience 1–2 years].*

According to the interviewees, climbing the installations was challenging, in particular when wearing survival suits and carrying heavy materials. Moreover, tasks involving the climbing of ladders, working at heights, or in forced postures on and inside the installations were regarded as physically demanding by both workers and experts:

*“There is a huge physical component [that comes with the job], with climbing and working in awkward positions and at heights, etc.” [expert, ID #4, offshore experience > 4 years].*

#### Transfer and access to installations

Transfer and access to the offshore installations were considered particularly demanding by the interviewees. Accessing the installations was perceived to be physically challenging:

*“Skipping up a ladder in a survival suit is certainly not everyone’s cup of tea.” [expert, ID #3, offshore experience > 4 years].*

Beyond that, it was also seen as a psychological challenge, since unstable weather conditions could increase accident risks during the transfer and lead to mental stress and tenseness:

*“In that kind of situation there is a definite feeling of tenseness.” [employee, ID #2, age 31–40 years, offshore experience 3–4 years].*

Furthermore, some workers reported to feel a certain form of social pressure to access the installations despite situations of marginal weather conditions, as they reported that they didn’t want to be regarded as the only ones expressing reservations to access the installations. Notably, the level of stress seemed to correspond with the workers’ previous experiences working under such circumstances, the less-seasoned workers experiencing more difficulties. Besides, interviewees reported that rough weather during transfer to the installations could cause seasickness among the workers, thereby further complicating the task:

*“The problem of seasickness became apparent. It's a very real problem, affecting many people.” [expert, ID #14, offshore experience > 4 years].*

### Job resources

#### Meaning of work

For many offshore employees and experts, the job was reported to be of very high personal meaning. For instance, to many of the interviewees, working offshore meant adventure, challenge, and freedom:

*“For me personally it’s still a great adventure.” [employee, ID #2, age 31–40 years, offshore experience 3–4 years].*

In particular, the large-scale idea behind the offshore wind industry – the green energy revolution – contributed substantially to the sensation of performing meaningful work. Many of the interviewees further perceived offshore work as being something new and special. The pioneer spirit of the growing offshore branch was often emphasized:

*“You’re not just an electrician in a small company that does routine house calls, rather you’re a pioneer – there are not many people who do this type of work.” [expert, ID #8, offshore experience > 4 years].*

#### Perception of safety

Employees and experts felt that safety had the highest priority for their work offshore. Most of the workers claimed to generally feel safe due to strict safety procedures and well-organized work processes. Employees reported feeling well prepared for emergencies and described the medical care offshore as being readily accessible. Furthermore, some of the workers highlighted that colleagues always kept an eye on each other:

*“You are not only responsible for yourself, but also for your colleagues. And that is true in everything you do.” [employee, ID #4, age 41–50 years, offshore experience > 4 years].*

#### Motivation and satisfaction

Both work motivation and job satisfaction were reported as being high among the interviewees:

*“They love their job out there. When they go to work and are assigned an exciting task, you can see the smiles light up their faces, they are really fired up for it.” [employee, ID #8, age 31–40 years, offshore experience 1–2 years].*

### Category 2: Work organization

All job demands and resources named in terms of the category ‘work organization’ are depicted in Table 4.

**Table 4:** Job demands and resources in terms of the work organization

Main category: Work organization	
**Job demands**	
work time	lengthy and tedious, overtime
time pressure	prevalent in specific work situations
waiting times, weather days	occurrence of waiting times, e.g. due to bad weather
workflow	occurrence of delays and modifications
cost pressure	high costs involved in offshore projects
work schedule	14 days offshore perceived as long
personnel	too few offshore personnel
communication on−/offshore	difficulties in the communication and information flow between colleagues onshore and offshore
*experts only:* emergency medical care	knowledge of limited treatment options and lengthy emergency routes
**Job resources**	
work time	still manageable in length
payment	attractive remuneration
waiting times, weather days	occasionally helpful for relaxation
workflow	well-structured and coordinated
work schedule	allows much free time onshore, suitable regularity
*experts only:* emergency medical care	readily accessible
*experts only:* medical check-ups	suitable regularity, important in its function

### Job demands

#### Work time

In general, offshore workers and experts described the daily 12-h shifts as lengthy and tedious. Interviewees stated that it was common to work a lot offshore:

*“I am here [offshore] anyway, what else is there to do? I may as well work.” [expert, ID #9, offshore experience 3–4 years].*

Some workers described a certain loss of sense of time, stating that the hours spent offshore sometimes seemed to fade into the background; it was *“just normal that you always work” [employee, ID #7, age 20–30 years, offshore experience 1–2 years].* Working night shifts and on call was perceived as being strenuous. Some employees noted difficulties in completing their tasks within the 12-h shifts, e.g. due to work time being invested in daily preparation, follow-up, or documentation. Working overtime was also described as demanding, being particularly prevalent among workers with planning and management responsibilities:

*“I was told from the very beginning that, 'yes, having to work overtime is fairly common here’ [at management level].” [employee, ID #12, age 20–30 years, offshore experience 1–2 years].*

#### Time pressure

Some of the interviewees mentioned time pressure as a job demand that especially appeared to arise during sudden weather changes or in emergency situations:

*“When you start work and then get the message from the client that, ‘bad weather is coming, we have to hurry, hurry, hurry’ – that's a form of psychological stress.” [employee, ID #18, age 31–40 years, offshore experience > 4 years].*

The feeling of time pressure seemed to depend on the project stage, being more pronounced in the construction phase of the wind parks, where a strict time schedule was specified.

#### Waiting times, weather days

Both employees and experts described waiting times offshore as burdensome. Although it was recognized that waiting times served the offshore workers’ own safety, many of the employees perceived their occurrence as paternalistic. Waiting times in the form of so-called ‘weather days’ (days when work was impeded by bad weather) were described as particularly strenuous. Such days were accompanied by increased feelings of discontentment among the workers, even resulting in sensations of cabin fever:

*“They want to work, not just hang around. It is almost unbearable! After a week stuck on a ship, you start to feel like a rat in a cage.” [employee, ID #4, age 41–50 years, offshore experience > 4 years].*

### Job resources

#### Work schedule

Although the 14/14-work schedule was regarded as demanding, it was also considered a substantial job resource. Employees expressed their satisfaction with being able to spend much free time onshore and in regular intervals throughout the year. To many of the employees, this fixed work schedule represented one of the main benefits of offshore work:

*“That’s an important factor for me, if not the most important factor. That I have a regular schedule and can say: OK, at that time, I’ll be away, but afterwards, I’ll be 100% at home.” [employee, ID #15, age 41–50 years, offshore experience 1–2 years].*

### Payment

Payment and related surcharges were described as main job resources and motives for working offshore:

*“Financially, offshore work is always a good decision. If I'm being honest, I would have to say that that is the main reason why I do it.” [employee, ID #5, age 31–40 years, offshore experience 1–2 years].*

### Category 3: Work environment

All job demands and resources named in terms of the category ‘work environment’ are depicted in Table 5.

**Table 5:** Job demands and resources in terms of the work environment

Main category: Work environment	
**Job demands**	
weather conditions	dependency on the weather, bad weather causes work interruptions, delays and waiting times
workplace design	confined spaces on platforms and installations
physicochemical factors	e.g. noise, vibrations, conditioned air, artificial light, chlorinated water
**Job resources**	
weather conditions	good weather offshore

### Job demands

#### Weather conditions

Both employees and experts highlighted the dependency on weather conditions as a relevant stressor of the offshore workplace. Seasonal complications (e.g. in the summer when temperatures were high, or in the winter when cold and rainy weather prevailed) were perceived as burdensome:

*“The weather conditions out there are relatively extreme. When the sun is shining, it shines intensely; when the wind is blowing, it blows very hard.” [expert, ID #16, offshore experience 3–4 years].*

According to the interviewees, unfavourable weather could result in work interruptions and delays of crew transfer, which could lead to negative feelings among the workers.

### Job resources

#### Weather conditions

On a positive note, good weather was regarded as a job resource. Employees reported to appreciate being able to enjoy good conditions during their lunch breaks and free time offshore. They particularly highlighted the *“fresh air and magnificent sunrises and sunsets” [employee, ID #16, age 31–40 years, offshore experience > 4 years].*

### Category 4: Social relations

All job demands and resources named in terms of the category ‘social relations’ are depicted in Table 6.

**Table 6:** Job demands and resources in terms of social relations

Main category: Social relations	
**Job demands**	
conflicts with colleagues	e.g. regarding work methods or differences in remuneration
international work environment	language barriers
**Job resources**	
social support	good comradeship and strong support from the team
international work environment	possibilities to improve language skills

### Job demands

Job demands regarding social relations were rarely described by the interviewees. For example, employees mentioned discussions with colleagues regarding work methods or differences in remuneration:

*“There are disputes about the fact that people receive varying compensation for the same jobs. I mean, it's loudly discussed why someone else earns more money than I do for the same type of work.” [employee, ID #16, age 31–40 years, offshore experience > 4 years].*

### Job resources

#### Social support

Both offshore employees and experts described the social support from colleagues and executives as one of the greatest resources of offshore work. In particular, employees reported strong ties as well as a sense of comradeship within the group, and highlighted the fact that new friendships developed quickly when working offshore:

*“It’s a really great thing that, after 14 days, you have 55 good friends here, with whom you enjoy spending your time.” [employee, ID #7, age 20–30 years, offshore experience 1–2 years].*

Some of the interviewees pointed out that the offshore business in Germany was still a manageable, relatively small market. Thus, there is a good chance that offshore colleagues will meet again over time in different projects, which was regarded positively:

*“We all keep saying, offshore is like a small village.” [employee, ID #4, age 41–50 years, offshore experience > 4 years].*

#### International work environment

Some of the employees described meeting international colleagues and improving their language skills as positive side effects of working offshore:

*“You meet so many new, interesting people from different countries.” [employee, ID #5, age 31–40 years, offshore experience 1–2 years].*

### Category 5: New forms of work

All job demands and resources named in terms of the category ‘new forms of work’ are depicted in Table 7.

**Table 7:** Job demands and resources in terms of new forms of work

Main category: New forms of work	
**Job demands**	
absence from home	absence from home, family, and friends
catering	too rich in calories, hearty, unhealthy
accommodation	confined spaces, cramped cabins, double cabins
opportunities for leisure activities and retreat	limited possibilities for sports, leisure activities, and retreat
means of communication	often location dependent or only partially available
**Job resources**	
free time at home	intensive time at home during free time onshore
catering	healthy food offers available
accommodation	sufficient in size
opportunities for leisure activities and retreat	various opportunities: exercising, watching movies, playing games, reading books, participating in social activities

### Job demands

#### Absence from home

Most of the workers considered the intermittent absences from home as one of the main challenges of their job, and feelings of homesickness were expressed by some workers. Such feelings seemed to increase when communication with the families and partners onshore was limited:

*“Generally, the men here are ‘tough guys’. But when the telephone for calling home doesn’t work, they are suddenly 12 years old again, get really homesick, and then they are useless.” [employee, ID #8, age 31–40 years, offshore experience 1–2 years].*

Offshore workers described that they found it particularly hard not being able to help their partners at home in their daily tasks. Missing pivotal moments in their children’s development and missing out of events onshore was also described as being difficult. The struggle of dealing with *“two different everyday lives” [expert, ID #8, offshore experience > 4 years]* – one onshore and one offshore – was highlighted. The level of burden experienced appeared to depend on the workers’ personality and their personal situations. Fathers, for example, reported to suffer more from the absences from home in comparison to non-fathers in a relationship and singles:

*"It also depends on whether you're married or single. All these younger, single people have no problem working four weeks straight. Those of us who are married, however, wish they could have some more family time." [employee, ID #4, age 41–50 years, offshore experience > 4 years].*

#### Catering

Catering offshore was regarded as extremely important by the interviewees for their well-being and work performance. However, some workers complained about certain aspects of the catering, e.g. that it was too rich in calories, too hearty, or consisted of too much meat:

*“There is meat, and then meat, and then even more meat.” [employee, ID #5, age 31–40 years, offshore experience 1–2 years].*

#### Accommodation

Depending on the specific type of accommodation, some interviewees described to sleep in cramped, uncomfortable cabins, causing a feeling of discomfort. Especially those required to share a double cabin reported to experience a lack of privacy. Furthermore, a few employees stated that the boundary between work and leisure time sometimes seemed to blur due to the shared or overlapping spaces of their offshore workplaces and living accommodations. However, other workers reported to have eventually been able to adjust to the particularity of working and living at the same place:

*“It's just how it is; where you work is where you live.” [employee, ID #3, age > 50 years, offshore experience 3–4 years].*

#### Opportunities for leisure activities and retreat

In general, employees and experts perceived the limited opportunities for retreat and leisure activities as demanding:

*“You actually just sit there for 14 days in this form of prison; you sit there in your cell and are very limited in your free-time activities.” [expert, ID #7, offshore experience 3–4 years].*

Employees particularly complained about the limited possibilities for sports and exercise. For instance, gyms (when available) were described as being too small and insufficiently equipped.

### Job resources

#### Free time at home

Employees reported positive effects of offshore work on their private and family life. They highlighted the fact that they were able to spend intensive time with their families and to actively engage in childcare during their free time periods onshore:

*“My son is now 7 months old. When we go to baby swimming lessons I am the only dad there, because all of the other dads are at work.” [employee, ID #6, age 20–30 years, offshore experience < 1 year].*

### Catering

Even though some employees complained about unhealthy food, the majority of the interviewees expressed their satisfaction with the catering. The experts were in particular of the opinion that offshore workers were provided with a great variety of high-quality food offers:

*“The food is healthy. It's like when you're staying in a good hotel.” [expert, ID #12, offshore experience 3–4 years].*

### Employees’ needs for improvement

In the interviews, we asked offshore employees about their needs for improvement regarding their working conditions. Few of the employees reported being satisfied with their current job situation and provided no recommendation for change. The majority of workers, however, suggested various needs for improvement. These are related to the work categories and depicted in Table 8. Most of the suggestions made by the employees fell under the categories of work organization and new forms of work. With regard to work organization, employees expressed a particular need for changes in the working hours:

**Table 8:** Employees’ needs for improvement

**Category 1: Work tasks**	
transfer and access to installations	promotion of greater awareness among the workers when accessing installations definition of maximum waiting times when accessing installations
**Category 2: Work organization**	
communication off−/onshore	promotion of a better mutual understanding and flow of information between colleagues offshore and onshore
personnel	increase in offshore personnel
workflow	definition of clear work processes
work time	changes in work tasks to avoid overtime work reduction of working hours (e.g. from 12 to 10 h) definition of break and leave regulations
work schedule	changes in work schedule (e.g. from 14 to 10 days offshore)
payment	increase in payment
medical care & check-ups	establishment of uniform international standards for check-ups
**Category 3: Work environment**	
workplace design	assurance of ergonomic workplace design
physicochemical factors	provision of room humidifiers in cabins to avoid dry air
**Category 4: Social relations**	
	–
**Category 5: New forms of work**	
private and family life	assurance of stable telephone and internet connections
accommodation	provision of single cabins of reasonable size
catering	arrangements with catering to improve healthy diet
opportunities for leisure activities and retreat	improvement of sports and leisure opportunities

*“I think it would make sense to reduce the 12-hour shifts. Maybe make them 10 hours... something along those lines.” [employee, ID #18, age 31–40 years, offshore experience > 4 years].*

Regarding new forms of work, they stressed the importance of stable telephone and internet connections, the improvement of leisure possibilities, and the provision of single cabins:

*“In order to ensure that everything runs smoothly, every employee who works out here should have access to a single cabin.” [employee, ID #7, age 20–30 years, offshore experience 1–2 years].*

For the category of social relations, there was no perceived need for improvement.

## Discussion

The aim of our study was to investigate the working conditions of employees in the German offshore wind industry and to identify areas in need of improvement. By conducting our interview study, we were able to gain important insights into these topics.

### Offshore employees’ working conditions

By drawing on the JD-R model [23, 25], we were able to classify a broad range of demands and resources of offshore work. In summary, employees and experts perceived most of the job demands to be under the categories of work organization and new forms of work, whereas the fewest demands where expressed for the category of social relations. In contrast, most of the job resources were found in the category of work tasks. Consistent with the JD-R model’s assumptions, we found that employees perceived job demands as hindering factors, whereas job resources were regarded as facilitating offshore work.

Interestingly, we found that some aspects were considered to be both a potential job demand *and* job resource, depending on the specific context (e.g. weather conditions: good weather was seen as a resource, and harsh weather was regarded as a demand). In the literature, offshore employees’ working conditions have been exclusively described unilaterally, either as a job demand *or* as a resource. In our study, we were able to achieve a further differentiation.

With respect to the work tasks, the hard physical work and challenging transfer and access to the installations were identified as job demands. This is consistent with certain considerations made for workers in the German [7–10] and international offshore wind industry [27], as well as in the offshore oil and gas industry [13, 17, 28]. Findings from the offshore wind industry indicate the most frequent incidents offshore to occur during the transport of people and material, including accessing wind farms and installations [27]. Despite this, the topic hasn’t received particularly much attention as a job demand for workers in the German offshore wind industry. Our result reinforces the relevance of this demand, being made further important by the fact that offshore workers may transfer to and from the installations several times per shift.

An important finding from our study is that the workers reported to feel safe at their place of work. Because the offshore workplace presents specific hazards, particular importance has been placed on safety concepts in the offshore wind industry [7] and other high-risk industries [29, 30]. Since it was found that risk perceptions present a stressor for offshore workers [17, 31] with potential negative impacts on their health [32], the strong perception of safety among our interviewees can be considered an important resource.

Demands associated with the work organization consisted, in part, of long working hours and shift work. This agrees with theoretical assumptions and empirical findings for employees in the German [7, 9, 10] and international offshore wind industry [14] as well as in the offshore oil and gas sector [13, 29, 33]. A relevant finding of our study is that waiting times and weather days represented a substantial job demand for the interviewees. There has been discussion concerning waiting times and the burden they represent in the form of work delays [9, 34]; however, waiting times haven’t been described as a burden in of themselves. This might be due to the fact that, until now, primary focus has been placed on demands regarding employees’ work situation, and not on the demand of *not* being able to work.

We found that the 14/14-work schedule, on the one hand, represented a particularly positive factor of offshore work for many interviewees. In studies from the offshore oil and gas sector, the work schedule has also been described as a potential benefit of offshore work, allowing the employees long periods of time off work [12, 29, 33]. However, preferences concerning work schedules have generally been found to vary across different age groups [35], such that our finding should be interpreted with caution. Our interviewees were relatively young, and older offshore workers might be less satisfied and able to cope with the 14/14-work schedule. Research indicates, for example, that aging workers cannot tolerate shift-work as well as their younger colleagues [36, 37], and that they have a higher need for recovery after work [38]. On the other hand, the work schedule was also perceived as demanding. In particular, our findings reinforce that being away from home represents a job demand for workers in the German offshore wind industry [7, 9]. Work-family-interface has also been described as a stressor for offshore oil and gas workers [13, 17, 31] and seafarers [20]; these workers tend to spend even longer periods of time away from home. Our findings show that some workers felt difficulties reconciling work and family life, supporting the notion that intermittent absences from home can contribute to work-family-conflicts for offshore wind industry workers [39]. In addition, we found workers to experience difficulty switching between their two social environments (offshore and onshore), an aspect that has also been revealed for offshore oil and gas workers [40, 41].

Job demands concerning the work environment were found to be in line with existing considerations for workers in the offshore wind industry, underlining unfavourable weather conditions and confined spaces at the workplace as demanding [7, 10, 27]. Such demands have similarly been discussed for offshore oil and gas workers [13, 17, 29] and seafarers [18].

In our study, conflicts with colleagues and language barriers were rarely described by either workers or experts. This finding is somewhat contradictory with theoretical considerations for workers in the German and international offshore wind industry, in which the limited social contact, conflicts with colleagues, and language barriers have been described [7, 9, 14, 27]. Likewise, in studies from the oil and gas sector and seafaring branch, a lack of social support [13, 18] and loneliness on board [20] have been reported. It should be noted that our finding only reflects the views of workers fluent in the German language; non-German speakers might encounter more difficulties in social interactions with German colleagues. However, results from other studies also support our finding of good social relations between offshore colleagues. For example, many workers in the offshore oil and gas industry were found to describe their colleagues as their second family [29], and social support was identified as a job resource with positive effects on offshore oil and gas workers’ health [15, 16, 32, 42, 43].

Remarkably, interviewees put special emphasis on job demands concerning new forms of work. Identified demands regarding the offshore accommodations (e.g. confined cabins, lack of privacy) and limited opportunities for leisure activities are in line with findings and theoretical considerations for workers in the offshore wind [7, 9] and oil and gas industry [17, 31]. Furthermore, our result that suitable catering was particularly important to the workers in our study agrees with findings from the offshore oil and gas industry [43–45].

Interestingly, we were able to identify additional job demands which have not been previously described in the literature, such as a perceived cost pressure at work. Employees felt that the perception of cost pressure was associated with time pressure (in the way that economic losses were anticipated when employees weren’t able to finish their work in time). Despite there being little discussion concerning cost pressure, the related aspect of time pressure has been described as a job demand in the offshore wind industry [9, 14, 27]. Another previously unknown demand revealed in our study was the limited access to means of communication. As limited chances for communication were described to provoke dissatisfaction among the workers, adequate means of communication seem to be essential for their well-being.

### Influencing factors regarding employees’ working conditions

We found that interviewees’ working conditions varied considerably, e.g. in terms of their specific work tasks, work contracts and salaries, as well as work and living environment (e.g. type of accommodation, catering, and opportunities for leisure activities). Moreover, the working conditions appeared to differ according to project phase (e.g. time pressure was reported to be more prevalent in the construction stage of the wind parks). These results are in line with findings from the offshore oil and gas industry, showing that, for example, sources of stress were specific to certain groups of offshore personnel with different work tasks and workplaces [31, 42, 46, 47]. In addition, employees perceived the level of burden of the job demands to varying extents, depending on their previous experiences in the offshore work environment or related industries. There was a tendency towards the perception of less job demands, the more experienced workers were. Furthermore, the workers’ personality and attitude seemed to play a role in their perceptions of job demands. Similarly, personality was found to be relevant for offshore oil and gas workers’ well-being, high extraversion and low neuroticism being associated with adaptability [46].

### Comparison of employees’ and experts’ perceptions

The views of offshore employees and experts mostly coincided with regards to job demands and resources. Observed differences mainly involved the emphasis they put on specific aspects. Experts, for example, spoke more often of demands related to heavy work equipment, the workplace design, or the emergency medical care. This finding should be interpreted in the context of offshore experts’ professions, since demands concerning the work equipment and workplace directly relate to experts’ occupational fields (e.g. health and safety). The fact that demands regarding emergency medical care were stressed by the experts is also reasonable, as they are directly involved in the design of offshore rescue concepts. In contrast, offshore workers emphasized demands associated with new forms of work, e.g. inconvenient accommodations and limited means of communication. Naturally, the living conditions offshore should be of greater importance to the workers in comparison to the experts who spend less time offshore. Regarding job resources, experts emphasized the medical care and medical check-ups, and spoke more positively about the living conditions offshore. In contrast, the scope of action, well-structured workflow, and international work environment were more in the forefront for the workers.

### Strengths and limitations

An important strength of our study is the fact that we conducted interviews with offshore workers *and* offshore experts. Therefore, we were able to incorporate different points of view and to contrast interviewees’ perspectives with each other. Workers and experts differed in their ages and years of experience in the offshore branch, which allowed us to incorporate a wider range of subjective perceptions. Moreover, we utilized rich descriptions of the results and direct quotes from the interviewees, which contributes to the trustworthiness of qualitative research results [48]. Another strength of our study stems from the fact that, during the data analysis process, the results were discussed thoroughly within the group of researchers involved in the study, which strengthened the transparency of the research process. In addition, the findings were compared with an appropriate theoretical framework (JDR-model), and empirical references [49]. Over time, the various job demands and resources were repetitively described by the interviewees, eventually reaching a point at which no new information were collected. Hence, it can be assumed that the 42 interviews we conducted were sufficient to achieve data saturation [50]. However, further quantitative studies are needed to generalize the results. Since our results are solely based on the views of persons directly related to the industry, it would be interesting to focus on external evaluations of the working conditions by involving independent authorities. Future studies should also investigate offshore workers’ health and study the impact of the working conditions on health-related outcomes. To date, many German offshore workers are relatively young. As the workers age, special focus should be placed on the question of whether or not the offshore workplace can be considered ageing-appropriate.

A restriction of our study is the fact that we conducted telephone interviews instead of face-to-face-interviews. Telephone interviews have generally been found to provide detailed, high quality data [51]; however, the asynchronous communication of place by telephone and subsequent reduction of certain social cues might have influenced the results [51, 52]. Moreover, the potential influence of the interviewer on interviewees’ responses as well as answer tendencies due to social desirability must be borne in mind. Some of the workers and experts were interviewed during their offshore assignments, while others were interviewed during their free-time periods onshore. The diverse geographical settings (work sector versus leisure sector) might also have influenced interviewees’ perceptions. Furthermore, interviewees were rather young in age and had relatively few years of offshore work experience. It might be assumed that our study could have revealed different results for older workers with more years of offshore experience. We only interviewed employees fluent in German and mostly spoke with male workers, so we could not account for the views of non-German speakers nor include gender perspectives in our findings. However, the male-dominated sample accurately reflects the predominance of male workers in the German offshore wind industry. Due to the qualitative nature of our study and the fact that we didn’t apply a statistical method to select interviewees, our sample cannot be considered as representative of the entire German offshore working population.

## Conclusions

To our knowledge, this is the first study to systematically analyse the working conditions and self-perceived needs of employees in the German offshore wind industry. Overall, our results can provide a basis for further quantitative research in order to generalize the findings. Moreover, the results can be utilized to develop needs-based interventions to improve the working conditions offshore. Since most of the needs for improvement expressed were in regard to the work organization and living conditions offshore, we highly recommend that these aspects be accounted for in the design of suitable interventions.

## Additional file

Additional file 1: Main and sub-categories for working conditions. (PDF 94 kb)
