# Supplementary material for: “It’s still a great adventure” – exploring offshore employees’ working conditions in a qualitative study
Source: J Occup Med Toxicol. 2017 Dec 8;12:35. doi: 10.1186/s12995-017-0179-0 (PMC5723037; doi:10.1186/s12995-017-0179-0)
Supplement: Additional file 1: — Main and sub-categories for working conditions. (PDF 94 kb) [file 12995_2017_179_MOESM1_ESM.pdf]

Additional file 1: Main and sub-categories for working conditions

	Job demands	Job resources
**Work tasks**	physical work<br>transfer and access to installations<br>accident risks<br>workloads<br>demanding work tasks<br>work equipment	meaning of work<br>perception of safety<br>motivation and satisfaction<br>challenging work tasks<br>skills and competencies<br>versatility of work<br>scope of action
**Work organization**	work time<br>time pressure<br>waiting times, weather days<br>workflow<br>cost pressure<br>work schedule<br>personnel<br>communication on-/offshore<br>*experts only:* emergency medical care	work time<br>payment<br>waiting times, weather days<br>workflow<br>work schedule<br>*experts only:* emergency medical care<br>*experts only:* medical check-ups
**Work environment**	weather conditions<br>workplace design<br>physicochemical factors	weather conditions
**Social relations**	conflicts with colleagues<br>international work environment	social support<br>international work environment
**New forms of work**	absence from home<br>catering<br>accommodation<br>opportunities for leisure activities and retreat<br>means of communication	free time at home<br>catering<br>accommodation<br>opportunities for leisure activities and retreat
